# Effects of Blue Light Exposure on Hepatic Inflammation and Gut Microbiota in Mice Consuming a High-Fat, High-Fructose Diet

**DOI:** 10.3390/nu18010164

**Published:** 2026-01-04

**Authors:** Wen-Chih Huang, Pei-Ni Lee, Wan-Ju Yeh, Wen-Chi Wu, Hsin-Yu Shih, Yi-Jen Chen, Hsin-Yi Yang

**Affiliations:** 1 Department of Anatomical Pathology, Far Eastern Memorial Hospital, New Taipei City 220, Taiwan; pathology.taipei@gmail.com; 2 Department of Nutrition, Taipei Hospital, Ministry of Health and Welfare, New Taipei City 24250, Taiwan; diet10096@tph.mohw.gov.tw; 3 Graduate Program of Nutrition Science, School of Life Science, National Taiwan Normal University, Taipei 11677, Taiwan; wandayeh@ntnu.edu.tw; 4 Department of Nutritional Science, Fu Jen Catholic University, New Taipei City 242, Taiwan

**Keywords:** blue light, liver, oxidative stress, inflammation, gut microbiota

## Abstract

**Background:** High-fat or high-fructose consumption may cause abnormal lipid accumulation in the liver, resulting in fatty liver disease, and the intervention of other stress factors may accelerate the progression of this condition. Many studies have demonstrated that long-term exposure to blue light may not only injure the eyes but also cause an increase in oxidative stress, which has been related to metabolic and gut microbiota disorders. However, current research on whether blue light exposure exacerbates fatty liver disease still remains limited. **Objective:** Therefore, the aim of this study is to investigate the effect of a high-fat, high-fructose diet combined with blue light exposure on fatty liver disease progression. **Method:** In the first part of the study, we observed that 16 weeks of blue light exposure alone did not achieve significant effects in the liver of male, female, or OVX mice. Therefore, in the second part, we fed ICR mice a high-fat, high-fructose (HFHF) diet to investigate the effect of simultaneous 16-week exposure to blue light. The mice were assigned to three groups, control group (C), HFHF diet group (H), and HFHF diet plus blue light exposure group (HB), to investigate the intervention of unhealthy diet composition and blue light exposure on hepatic oxidative and inflammatory makers and gut microbiota composition. **Results:** The results showed that exposure to blue light exacerbates oxidative stress (hepatic MDA, *p* < 0.009), and inflammatory damage (lobular inflammation score, *p* < 0.0001; hepatic TNF-α, *p* = 0.0074) caused by an HFHF diet, but this mechanism is not mediated by the TLR4 signaling pathway. Furthermore, exposure to blue light may also partially affect the composition of the gut microbiota. **Conclusions:** The results of the study suggested that under unhealthy dietary conditions, long-term blue light exposure may be one of the risk factors accelerating the progression of fatty liver disease.

## 1. Introduction

**Figure 1:**
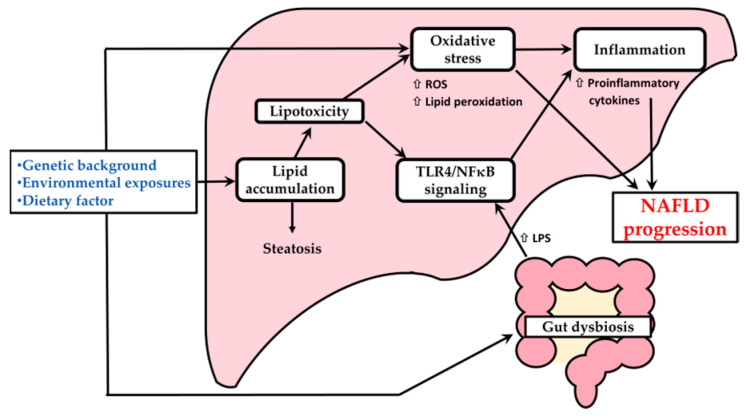
Multiple-hit hypothesis for the pathogenesis of NAFLD. NAFLD, nonalcoholic fatty liver disease; ROS, reactive oxygen species; TLR4, Toll-like receptor 4; NF-κB, nuclear factor kappa-light-chain-enhancer of activated B cells; LPS, Lipopolysaccharide.

Changes in modern lifestyles are important factors contributing to many metabolic disorders. The excessive intake of lipids and simple carbohydrates in the diet, combined with oxidative stress caused by surrounding factors, could result in metabolic abnormalities and chronic inflammatory damage to tissues throughout the body. The prevalence of nonalcoholic fatty liver disease (NAFLD) has shown a rapid increase over the past two decades, and the age of onset has started to show a trend toward younger people, making it an important public health issue that cannot be ignored [1]. The pathogenesis of NAFLD is a complex process that can potentially progress from simple hepatic lipid accumulation to mild or severe inflammation, ultimately resulting in tissue damage and fibrosis. In recent years, the multiple hit hypothesis has been the most reported, indicating that NAFLD may be caused by the interaction of various factors, including many genetic and external environmental factors, the release of pro-inflammatory substances, and the influence of the gut-liver axis, all of which may play roles in the progression of NAFLD [2,3] (Figure 1).

The increase in oxidative stress is a key factor accelerating the progression of NAFLD, and it also represents the result of an imbalance between reactive oxygen species (ROS) and the body’s antioxidant defense system. Nuclear factor erythroid 2-related factor 2 (Nrf2) is a transcription factor that regulates redox reactions in the body to modulate oxidative stress in vivo, and it can activate the downstream anti-oxidative system when the internal environment changes and ROS increases, achieving the effect of regulating oxidative stress and reducing lipid peroxidation [4]. Gut dysbiosis may be another important factor in the progression of NAFLD. When excessive endotoxins in the intestinal lumen pass through the intestinal mucosa into the blood circulation, caused by diet or other environmental factors, they flow into the liver via the portal vein, thereby activating the Toll-like receptor 4 (TLR4) signaling pathway; this results in the activation of nuclear factor kappa-light-chain-enhancer of activated B cells (NF-κBs) via the downstream myeloid differentiation primary response gene 88 (MyD88) pathway, increasing the secretion of pro-inflammatory cytokines such as tumor necrosis factor (TNF)-α and interleukin (IL)-1β, resulting in inflammatory reactions in the liver. Many experiments have reported that long-term high-fat or high-fructose diets may lead to an imbalance in the gut microbiota and impair normal physiological functions [5,6].

Additionally, light-emitting diodes (LEDs) have been widely used in various indoor lighting equipment in recent years. Because LED is a light source rich in blue light, which is more likely to penetrate eye tissue and reach the retina, high-intensity or prolonged exposure may cause tissue damage due to its short wavelength and high energy [7]. Due to the global COVID-19 pandemic, the public has become more accustomed to using online media for learning or working, which has also indirectly altered lifestyles and increased the time of exposure to blue light. Therefore, the harm that excessive exposure may cause to physiology is also very worthy of attention. In addition to the damage to eye tissue, some studies have begun to find that blue light exposure may also increase oxidative stress and inflammatory reactions in the body, and even affect the composition of the gut microbiota, thereby exacerbating metabolic disorders and other tissue damage [8,9,10]. However, current research on the relationship between blue light exposure and diet-induced NAFLD is still quite limited. Therefore, this study aims to clarify the potential effects of long-term exposure to blue light on hepatic injury. Firstly, we investigated whether exposure to blue light alone would affect liver health in mice of different genders and ovariectomy (OVX) status. Secondly, we further evaluate the effects of blue light exposure on hepatic lipid accumulation, oxidative stress, inflammatory responses, and gut microbiota in mice consuming an unhealthy, high-fat, high-fructose diet.

## 2. Materials and Methods

### 2.1. Experimental Design

**Figure 2:**
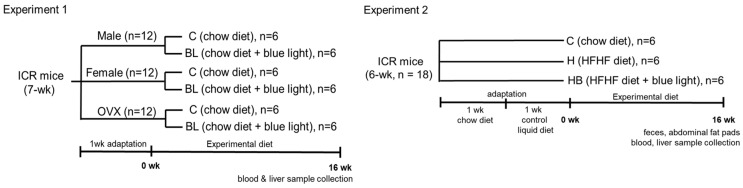
Experimental designs and procedures for Experiment 1 and Experiment 2.

Experiment 1: Seven-week-old male ICR mice were purchased from BioLASCO Taiwan Co., Ltd. (Yilan, Taiwan), comprising 12 males, 12 females, and 12 ovariectomized mice. All study protocols were reviewed and approved by the Institutional Animal Care and Use Committee of the National Taiwan Normal University (No. 114007 and 114013). The environmental conditions for the mice were maintained in a room at 22 °C, 55% humidity, and a 12 h light/dark cycle. After 1-week adaptation, the mice were randomly assigned to a control group or a BL group, in which the mice were exposed to blue light (465 nm BL LED light, Philips, Amsterdam, The Netherlands) at an intensity of 0.8 μW/cm^2^ (37.7 lux) for 6 h per day as previously reported, for a 16-week experiment [11]. All mice were fed a standard rat chow diet (Rodent Laboratory Chow 5001, Purina Mills, St. Louis, MO, USA) throughout the experimental period with free access to food. Body weight and food intake were recorded, and the mice were anesthetized with isoflurane and euthanized at the 16th week. Liver tissues were collected for further analysis. The experimental design is shown in Figure 2.

Experiment 2: Eighteen 6-week-old male ICR mice were purchased from BioLASCO Taiwan Co., Ltd. (Yilan, Taiwan). All study protocols were reviewed and approved by the Institutional Animal Care and Use Committee of the National Taiwan Normal University (No. 112027). The environmental conditions for the mice were maintained in a room at 22 °C, 55% humidity, and a 12 h light/dark cycle. The mice were fed a standard rodent chow diet (Rodent Laboratory Chow 5001, Purina Mills) for 1 week and then switched to a control liquid diet based on AIN93M for an additional 1 week to facilitate adaptation. Then, during the 16-week experimental period, the H and HB groups of mice were fed a high-fat, high-fructose (HFHF) diet [12], with some modifications using fructose instead of dextrin, while the C group of mice was fed a control liquid diet (Table 1). The mice in the HB group were exposed to blue light under the conditions described in Experiment 1. Body weight and food intake were recorded. At the end of the study, fresh fecal samples were collected from 4 randomly chosen mice in each group for microbiota analysis. Then, the mice were anesthetized with isoflurane and euthanized at the 16th week. Abdominal epididymal, perirenal, and mesenteric fat pads were collected and weighed. Blood and liver tissues were collected for further analysis.

### 2.2. Blood Analysis

The mice were sacrificed after an 8 h fasting, and we collected blood from the inferior vena cava. Serum was collected after centrifugation (1200× *g* at 4 °C for 15 min) for analyzing glucose, total cholesterol (TC), triglycerides (TGs), HDL-cholesterol (HDL-C), LDL cholesterol (LDL-C), aspartate transaminase (AST), alanine transaminase (ALT) and creatinine levels, all of which were analyzed using an autoanalyzer (Roche 110 Modular P800; Diamond Diagnostics, Holliston, MA, USA).

### 2.3. Liver Sample Analysis

For pathohistological analysis, the resected rat liver sections were fixed in formaldehyde (10% [*v*/*v*]) and then stained with hematoxylin and eosin. Tissue morphological changes were examined by a pathologist, and fat accumulation and damage were scored according to Brunt’s method [13]. The NAFLD activity scores (NASs) were calculated as the sum of scores for lipid accumulation (steatosis) degree, (lobular) inflammation severity, and hepatocellular ballooning degree. Immunohistochemistry was performed on the BenchMark Ultra machine (Ventana Inc., Tucson, AZ, USA). Then, 4-μm-thick sections were obtained from each paraffin block and mounted on positively charged glass slides for immunostaining with anti-NF-κB (Cell Signaling Technology, Inc., Danvers, MA, USA) and anti-NRF2 (Proteintech Group, Inc., Rosemont, IL, USA, 16396-1-AP) antibodies. Slides were pre-treated for antigen retrieval in Benchmark Ultra using the Ultra Cell Conditioning 1 reagent (Ventana Medical Systems, 06414575001) for Nrf2 (16 min) and Ultra Cell Conditioning 2 reagent (Ventana Medical Systems, 06414575001) for NF-κB (92 min). Dilutions of 1:200 for NF-κB and 1:400 for NRF2 were used. The immunostaining was performed using a Ventana BenchMark Ultra and OptiView DAB IHC Detection Kit system. The presence of a brownish color in the immunohistochemistry staining of NF-κB and NRF2 was considered positive.

To measure hepatic lipid levels, samples were homogenized and extracted using a chloroform/methanol mixture [12]. Triglyceride and cholesterol concentrations were then determined using commercial kits (Randox TR210 and Fortress BXC0271, Antrim, UK). In addition, liver tissues were homogenized in a buffer (50 mM Tris-HCl, 150 mM NaCl, 1% NP-40 (Sigma-Aldrich, I8896, St. Louis, MO, USA), and 0.1% sodium dodecyl sulfate (Bio-Rad Laboratories, 1610416, Hercules, CA, USA)) containing a protease inhibitor (Roche, 04693116001, Mannheim, Germany). After centrifugation, the supernatant was collected for further analysis. Lipid peroxidation marker malondialdehyde (MDA) levels were determined spectrophotometrically using the thiobarbituric acid reactive substances assay [14]. TNF-α and IL-1β, concentrations were measured using commercial ELISA kits (Mouse TNF-α DuoSet ELISA, DY-410-05; Mouse IL-1β/IL-1F2 DuoSet ELISA, DY-401-05, R&D Systems, Minneapolis, MN, USA) and adjusted by protein concentration measured with a Bio-Rad protein assay dye (Bio-Rad Laboratories, 5000006).

Western blotting was utilized to analyze the expression of proteins involved in the hepatic TLR4 pathway. Samples were initially prepared by homogenizing them in a lysis buffer (50 mM Tris-HCl, 50 mM NaCl, 1% NP-40, 0.1% SDS) supplemented with protease inhibitors. Following centrifugation, 30 μg of supernatant protein was loaded onto an 8% gel made with SDS-polyacrylamide (1610158, Bio-Rad Laboratories) for separation, after which the proteins were transferred to a polyvinylidene difluoride (PVDF) membrane. The membrane was initially blocked by incubation in non-fat milk to prevent the non-specific binding of antibodies. After washing with PBS/Tween-20, the membrane was combined with an anti-Toll-like receptor 4 (TLR4) antibody (monoclonal antibody to TLR4, IMG-5031A, Novus Biologicals, Centennial, CO, USA), anti-myeloid differentiation primary response 88 (MyD88) antibody (MyD88, D80F5 rabbit mAb, Cell Signaling Technology), and anti-TIR-domain-containing adapter-inducing interferon-β (TRIF) antibody (TRIF/TICAMI antibody, N8120-13810, Novus Biologicals) and secondary antibodies (horseradish peroxidase [HRP] donkey anti-rabbit immunoglobulin G [IgG] antibody 406401; HRP goat anti-mouse IgG antibody 405306, Biolegend, San Diego, CA, USA). After washing, the membranes were treated using a chemiluminescence detection system (PerkinElmer, Waltham, MA, USA) to visualize the immune complexes. The resulting bands were then quantified using the BioSpectrum AC image system, UVP Visionwork LS software, and Image-Pro Plus 4.5 (Media Cybernetic, Rockville, MD, USA). β-actin (Proteintech, 20536-1-AP, IL, USA) served as the total protein loading control. Data are ultimately reported as the relative proportion of the protein compared to the control protein.

### 2.4. Fecal Analysis

We randomly selected fresh fecal samples and extracted DNA with a QIAamp DNA Stool Mini Kit (Qiagen, Germantown, MD, USA) according to Godon’s method [15]. Illumina HiSeq Sequencing (Illumina, San Diego, CA, USA) was performed on the amplified 16S rDNA V3-V4 regions to generate raw data post-extraction. High-quality sequences (effective tags) were obtained by filtering chimeras using UCHIME and then clustering them at a sequence identity of greater than 97% [16]. To normalize for sampling depth differences across samples, abundance data were retrieved from operational taxonomic units (OTUs), ensuring that all samples met a minimum sequence count. Alpha and beta diversity were analyzed as previously reported, and the *Firmicutes*-to-*Bacteroidetes* (F-B) ratio was also calculated [17]. The Shannon index was implemented to measure alpha diversity, whereas beta diversity was visualized through PCA based on OTU abundance. Taxonomical profiling was conducted at the phylum, class, family, and genus levels to assess relative abundance distributions.

### 2.5. Statistical Analysis

GraphPad Prismatic 10.4.0 (GraphPad Software, San Diego, CA, USA) and Microsoft Excel 2016 (Microsoft Corporation, Redmond, WA, USA) were used for statistical analysis and figure preparation, with values expressed as the mean and standard deviation (SD). Two-way analysis of variance (ANOVA) and Tukey’s multiple range test were performed to compare data among groups following Experiment 1. Two-way ANOVA was used to evaluate the main effects of sex/OVX status (male, female without OVX, and female with OVX) and light exposure (with or without blue light exposure), as well as their interaction. One-way ANOVA and Tukey’s multiple range test were performed to compare data among groups following experiment 2. Homogeneity of variances was assessed using the Brown-Forsythe test before conducting the ANOVA. To identify variations in microbial composition, the Wilcoxon rank-sum test was employed to detect absolute abundance shifts among the top 10 significantly different genera. Furthermore, the relative abundance of five key genera was compared across groups using STAMP analysis. A *p* value < 0.05 was considered significant. We determined the required sample size using G*Power 3.1.9.7 for a three-group one-way ANOVA comparison. Targeting a statistical power of 0.80 and an alpha level of 0.05 and considering the hepatic TG level as the primary outcome, the analysis showed that a minimum of 6 animals per group was required to detect an effect size (Cohen’s f) of 0.75.

## 3. Results

### 3.1. Effects of Blue Light Exposure on Mice of Different Sexes and Physiological States

#### 3.1.1. Effects of Blue Light Exposure on Weight and Food Intake in Mice of Different Physiological Conditions

In Experiment I, we found that although male mice were larger at the end of the experiment, long-term blue light exposure under a normal diet did not affect body weight, food intake, or liver weight in male, female, or OVX mice (Table 2).

#### 3.1.2. Effects of Blue Light Exposure on Hepatic Oxidative Stress and Inflammation in Mice of Different Physiological Conditions

**Figure 3:**
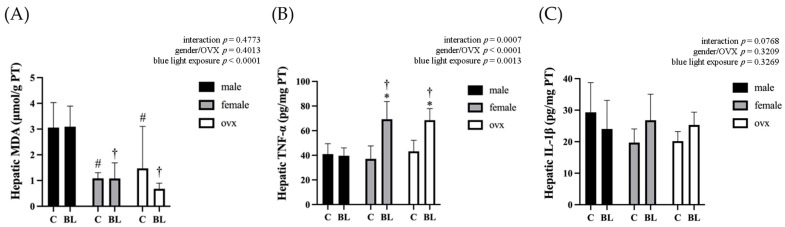
Hepatic malondialdehyde and inflammatory cytokine concentrations at the end of the experiment. (**A**) Hepatic MDA in male/female/OVX mice. (**B**) Hepatic TNF-α in male/female/OVX mice. (**C**) Hepatic IL-1β in male/female/OVX mice. Values represent means ± SD; *n* = 6 per group. Data were analyzed via two-way ANOVA followed by Tukey’s multiple range test analysis. * Significantly different from the control group of the same sex (C vs. BL, *p* < 0.05). ^#^ Significantly different from the C group of females or OVX compared with the male control group (C (male) vs. C (female), C (male) vs. C (OVX), *p* < 0.05). ^†^ Significantly different from the BL group of females or OVX compared with the male BL group (BL (male) vs. BL (female), BL (male) vs. BL (OVX), *p* < 0.05). C, control group; BL, blue light exposure group. MDA, malondialdehyde. TNF-α, tumor necrosis factor-α; IL-1β, interleukin-1β.

Further analysis of the hepatic oxidative and inflammatory markers (Figure 3) revealed that although the liver MDA concentration in males was higher than in females and OVX mice, blue light intervention showed no significant effect. In terms of inflammatory response, hepatic TNF-α levels exhibited a significant interaction between sex/OVX status and blue light exposure, with a significant increase observed only in female and OVX mice. However, no significant inflammatory response was detected in male mice. The results suggested that in the absence of other influencing factors, the blue light exposure duration may not have a significant effect on liver damage in the animals. Therefore, in Experiment 2, we further subjected the mice to an HFHF diet.

### 3.2. Effects of Blue Light Exposure on Mice Fed with a HFHF Diet

#### 3.2.1. Effects of HFHF and Blue Light Exposure on Weight, Food Intake and Biochemical Parameters

In Table 3, we found no difference in the average daily caloric intake among the three groups during the experiment, and there is also no significant difference in final body weight. The HB group exhibited significantly lower HDL-C levels and significantly higher hepatic function index ALT compared to the C group, but there was no difference in the liver weight or abdominal white adipose tissue weight to body weight ratio compared to the C group.

#### 3.2.2. Effects of HFHF and Blue Light Exposure on Hepatic Oxidative Stress and Inflammation

**Figure 4:**
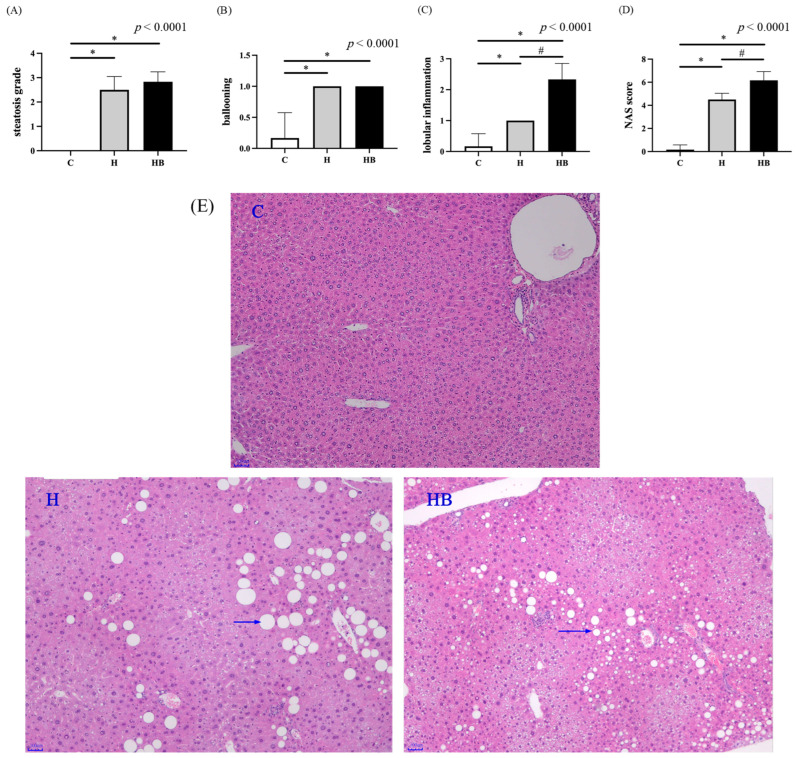
Hepatic histopathological analysis at the end of the study. (**A**) Steatosis score, (**B**) ballooning score, (**C**) lobular inflammation score, (**D**) NAS score, and (**E**) images of the hematoxylin and eosin–stained liver sections (magnification, 40×. Blue arrows indicate fatty change). Values represent means ± SD; *n* = 6 per group. Data were analyzed via one-way ANOVA followed by Tukey’s multiple range test. * Significantly different from the control group (C vs. H or HB, *p* < 0.05). ^#^ Significantly different from the high-fat diet group (H vs. HB, *p* < 0.05). C, control diet; H (HFHF), high-fat-high-fructose diet; HB, HFHF diet + blue light exposure group. NAS score, non-alcoholic fatty liver disease activity score was calculated as the sum of scores for lipid accumulation (steatosis) degree, (lobular) inflammation severity, and hepatocellular ballooning degree.

The H&E sections were used to observe histopathological changes in the liver at the end of the experiment (Figure 4). The control group shows normal liver parenchyma. However, observation of the H group revealed a picture of macrovesicular and microvesicular steatosis. Focal lobular inflammation and hepatocyte ballooning degeneration were also observed. In addition, the HB group exhibits more pronounced lobular inflammation and a higher NAS score.

**Figure 5:**
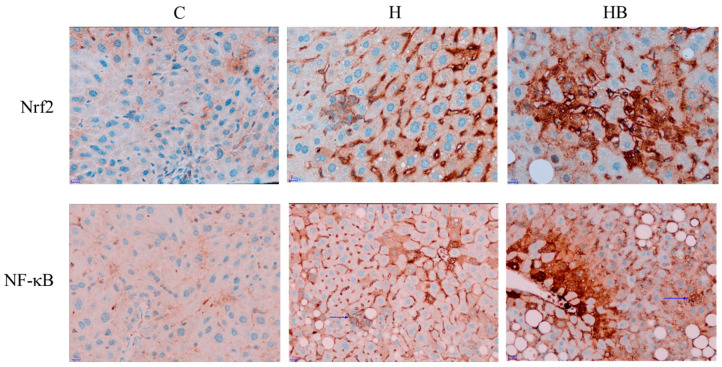
Immunohistochemical staining of Nrf2 and NF-κB (magnification, 200× and 100×. Blue arrows indicate lobular inflammation). C, control diet; H (HFHF), high-fat-high-fructose diet; HB, HFHF diet + blue light exposure group. Nrf2, nuclear factor erythroid 2-related factor 2; NF-κB, nuclear factor kappa-light-chain-enhancer of activated B cells.

To understand the potential effects of the HFHF diet and blue light exposure on oxidative stress and inflammation, we first use the immunochemical staining method to observe the hepatic Nrf2 and NF-κB expression. As shown in Figure 5, the C group shows no Nrf2 and NF-κB staining, while the H group shows focal low-to-moderate cytoplasmic expression of Nrf2 and NF-κB in hepatocytes, and chronic inflammatory cells are positive for Nrf2 and NF-κB. In the HB group, the Nrf2 and NF-κB stains demonstrate moderate to high expression in the cytoplasm of hepatocytes in non-alcoholic steatohepatitis. Focal nuclear staining is observed, indicating Nrf2 and NF-κB translocation into the nuclei.

**Figure 6:**
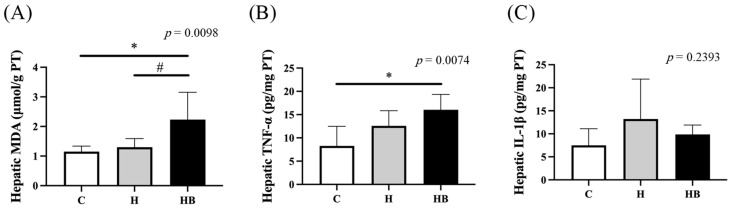
Hepatic malondialdehyde (**A**), pro-inflammatory cytokine TNF-α (**B**) and IL-1β (**C**) concentrations at the end of the study. Values represent means ± SD; *n* = 6 per group. Data were analyzed via one-way ANOVA followed by Tukey’s multiple range test. * Significantly different from the control group (C vs. H or HB, *p* < 0.05). ^#^ Significantly different from the high-fat diet group (H vs. HB, *p* < 0.05). C, control diet; H (HFHF), high-fat-high-fructose diet; HB, HFHF diet + blue light exposure group. MDA, malondialdehyde; TNF-α, tumor necrosis factor-α; IL-1β, interleukin-1β.

Therefore, we further analyze the related biomarkers in the liver tissue (Figure 6). We found that the HB group had higher hepatic MDA and proinflammatory cytokine levels than the C group, and exhibited higher MDA and IL-1β concentrations than the H group, indicating that blue light exposure may cause further liver damage.

#### 3.2.3. Effects of HFHF and Blue Light Exposure on the TLR4 Pathway and Gut Microbiota

**Figure 7:**
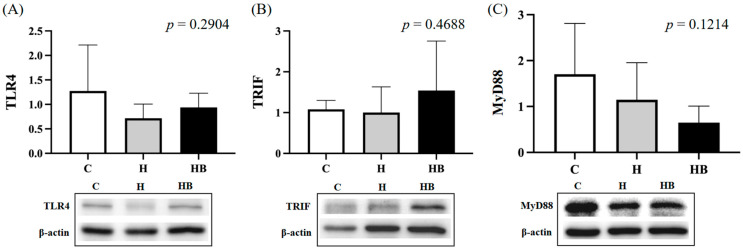
Hepatic protein expression of the TLR4 signaling pathway at the end of the study. (**A**) TLR4, (**B**) TRIF, and (**C**) MyD88 protein expression levels in the liver were analyzed via Western blot and normalized to β-actin. Values represent means ± SD; *n* = 6 per group. Data were analyzed by one-way ANOVA followed by Tukey’s multiple range test analysis. C, control diet; H (HFHF), high-fat-high-fructose diet; HB, HFHF diet + blue light exposure group. TLR, Toll-like receptor; TRIF, TIR-domain-containing adapter-inducing interferon-β; MyD88, myeloid differentiation primary response protein 88.

The TLR4 pathway plays an important role in the gut-liver axis. In the analysis of hepatic TLR4 pathway, we found no difference in TLR4, MyD88 and TRIF expression between groups (Figure 7).

**Figure 8:**
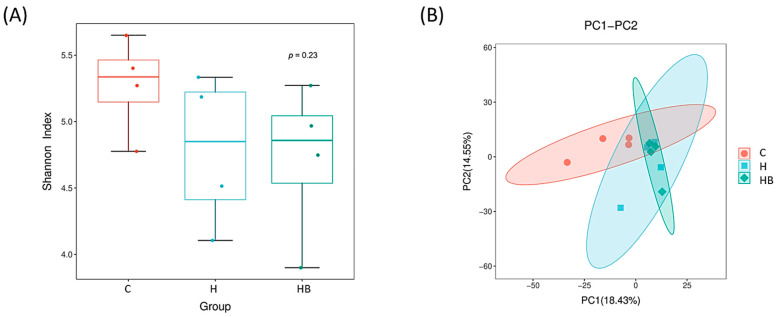
PCA for species-level differences in fecal microbiota. (**A**) Alpha diversity. (**B**) Beta diversity. Alpha diversity was assessed by the Shannon diversity index; data are presented as box plots, and statistical significance was evaluated using the Wilcoxon rank-sum test. Beta diversity visualized by principal component analysis (PCA) based on OTU abundance profiles. Each point represents an individual mouse fecal sample, and the percentages on the axes indicate the proportion of variance explained by each principal component. C, control diet; H (HFHF), high-fat-high-fructose diet; HB, HFHF diet + blue light exposure group.

To understand the differences in gut microbiota composition among groups, we randomly selected four mice from each group and collected fresh feces for next-generation 16S rRNA sequencing. First, we compared alpha diversity using the Shannon Index (Figure 8A) and observed that, although group C showed a trend of higher species richness compared to the other two groups, there were no significant differences among the three groups. We then performed beta diversity analysis using principal component analysis (PCA) to compare differences in species composition among the different groups (Figure 8B). We observed that the microbial community composition of the H and HB groups is more similar, while the C group differs more significantly compared to the other groups. These results are consistent with previous research findings [18], which suggest that an HFHF diet (H group) may affect species richness and composition compared to a balanced diet. Additionally, the blue light intervention exhibits few differences in the H group with respect to both alpha and beta diversity.

**Figure 9:**
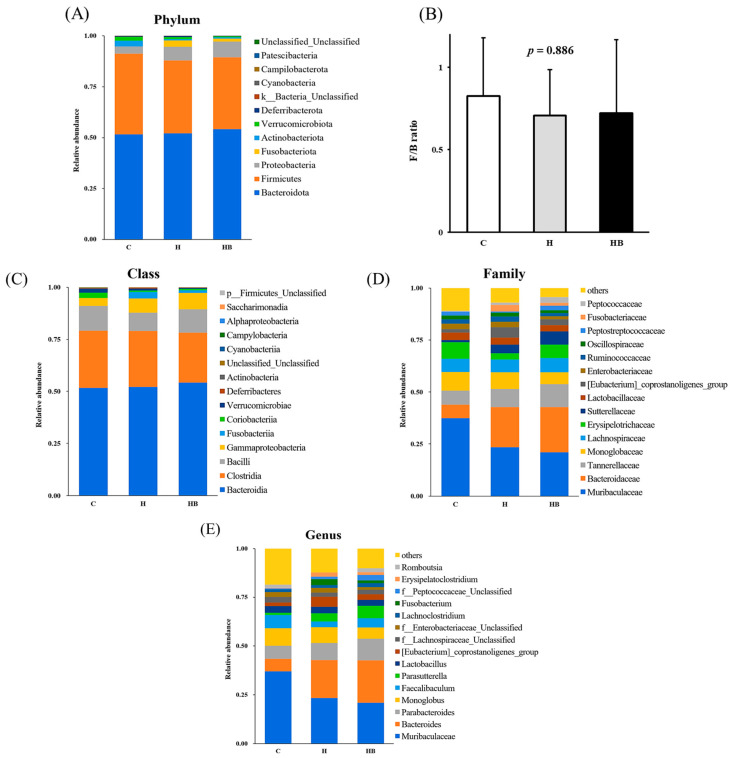
Relative abundance of bacterial communities in mice under different diets (*n* = 4). (**A**) Top 10 relative abundance of microbial species at the phylum levels in the feces of mice; (**B**) *Firmicutes*/*Bacteroidetes* (F/B) ratio; (**C**) class analysis; (**D**) family analysis; (**E**) genus analysis. F/B ratios are presented as mean ± SD, and statistical significance was evaluated using one-way ANOVA. C, control diet; H (HFHF), high-fat-high-fructose diet; HB, HFHF diet + blue light exposure group.

We also conducted comparative and abundance analysis of the microbial community species composition among different groups using taxonomic classification. In the comparison classified by phylum (Figure 9A), the three most abundant phyla were *Bacteroidota*, *Firmicutes*, and *Proteobacteria*; however, there was no significant difference in their proportions among the three groups. When calculated as the F/B ratio, there was also no significant difference among the three groups (Figure 9B). In the comparison classified by class, the three most abundant classes were Bacteroidia, Clostridia, and Bacilli; however, there were no significant changes in their proportions among the three groups (Figure 9C). In the comparison classified by family (Figure 9D), the three most abundant families were *Muribaculaceae*, *Bacteroidaceae*, and *Tannerellaceae*, and group C exhibited a higher trend in *Muribaculaceae* compared to the other two groups (*p* = 0.071) and a lower trend in Bacteroidaceae (*p* = 0.065), while Tannerellaceae showed no significant difference among groups (*p* = 0.510). In the comparison classified according to the genus, the most abundant genera were *Muribaculaceae*, *Bacteroides*, and *Parabacteroides*, of which group C showed a higher trend in *Muribaculaceae* compared to the other two groups (*p* = 0.075) and a lower trend in *Bacteroides* (*p* = 0.065) (Figure 9E).

**Figure 10:**
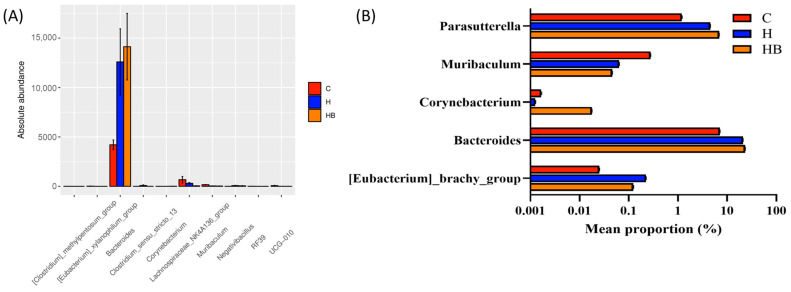
Differential species analysis of mouse gut microbiota. (**A**) Wilcoxon rank-sum test analysis showed the absolute abundance changes in the top 10 significantly different genera among the groups. (**B**) STAMP analysis showed the relative abundance of five significantly different genera, of which Bacteroides, Corynebacterium, and Muribaculum showed significant differences in both analytical methods. C, control diet; H (HFHF), high-fat-high-fructose diet; HB, HFHF diet + blue light exposure group.

In addition, we utilized the Wilcoxon rank-sum test (Figure 10A) and STAMP analysis (Figure 10B) to investigate the differences in gut genera among the different groups. Wilcoxon analysis revealed that the absolute abundance of *Bacteroides* was significantly higher in group H compared to group C, and it increased slightly in the blue light high-fat group (HB). *Muribaculum* also showed an increasing trend in the H and HB groups, and the abundance of *Corynebacterium* was slightly lower in the H and HB groups than in the C group. STAMP analysis further verified the above results, showing that five genera, *Parasutterella*, *Muribaculum*, *Corynebacterium*, *Bacteroides*, and [*Eubacterium*]_brachy_group, had significant differences among the three groups. The relative abundance of *Parasutterella* and *Bacteroides* was higher in the HB group, suggesting that blue light exposure may further promote the growth of these genera in an HFHF dietary background, and *Corynebacterium* exhibited a similar trend. Conversely, *Muribaculum* had the highest relative abundance in the C group and was relatively lower in the H and HB groups, while [*Eubacterium*]_brachy_group was highest in the H group, followed by the HB group. The results from the Wilcoxon and STAMP analyses demonstrate that the HFHF diet primarily resulted in an increase in *Bacteroides* and *Parasutterella* and a decrease in *Muribaculum*, while blue light exposure under high-fat conditions further influenced changes in *Corynebacterium*. Therefore, exposure to blue light and dietary factors may synergistically regulate the structure of the gut microbiota.

## 4. Discussion

Studies have proposed various hypotheses to explain the pathogenesis of fatty liver disease progression and the related metabolic disorders. Currently, the most widely accepted hypothesis of fatty liver disease’s pathological etiology is that it may be caused by the interaction of multiple factors, known as the multiple hit hypothesis. Therefore, for fatty liver disease induced by unhealthy dietary patterns, in addition to modifying eating habits, effectively controlling external environmental factors is crucial to reduce pro-oxidative reactions, the release of pro-inflammatory substances, and the potential impact of the gut-liver axis, all of which play quite an important role [2]. Recent studies have found that irregular light exposure may be important in modulating lipid metabolism in vivo, and the study by Guan et al. indeed revealed that, compared to green light and white light, 24 h continuous blue light exposure may increase oxidative stress and liver damage in the body by disrupting biological rhythm [19]. To simulate the current reality that might occur in everyday life, in this experiment, we adopted a long-term (16 weeks) exposure of 6 h per day. Since our previous study has already confirmed that this type of exposure, combined with an unhealthy diet, indeed causes visual damage [10], we further investigated potential effects on other body tissues, beyond the eyes.

In this study, we utilized animals of different genders and physiological conditions for research and found that, under a normal diet, blue light exposure only had a slight effect on liver TNF-α in female animals. However, when combined with a high-fat, high-fructose diet, we observed that blue light indeed increased the damage caused by an unhealthy dietary pattern. Modern lifestyles involve extensive exposure to artificial light sources and technological products, which increase the eye’s exposure to light and blue light. Some research suggests that blue light can accelerate retinal aging and may be a contributing factor to age-related macular degeneration [20]. However, besides affecting eye and retinal function, in 2013, the American Medical Association also suggested that nighttime exposure to light sources, such as electric lights or other electronic products with light sources, may increase the risk of many diseases, including cancer, obesity, diabetes, and psychiatric disorders, by disrupting the circadian rhythm [21]. Another study also found a positive correlation between greater exposure to artificial light at night and the incidence of obesity among middle-aged and older adults in the United States [22].

In animal experiments, mice housed in a 24 h light exposure environment showed obvious signs of fatty liver after 8 weeks compared to mice under a normal light-dark cycle [23]. In a study where rats were divided into two groups, one receiving a normal diet and the other a high-fat diet, they were housed under light-dark cycles and continuous light exposure for 12 weeks, respectively, to observe the effects on indicators related to fatty liver disease. Their results showed that continuous light exposure led to abnormal blood glucose and lipid concentrations in the animals, and feeding a high-fat diet exacerbated these abnormalities, resulting in conditions such as insulin resistance and steatohepatitis [24]. Recently, studies have further compared the effects of specific wavelengths of light on physiological functions. Blue light is a short-wavelength light with relatively high energy in the visible spectrum. Currently, we frequently use LEDs for lighting, mobile phones, computer liquid crystal displays, and tablets, among other applications [25]. However, the blue light power of white LED lights used for daily illumination is much higher than that of traditional light sources such as incandescent or fluorescent lamps [26]. This is because white light is ultimately created by combining LED blue light with excited yellow phosphor, and it indeed contains a strong blue light band (450–470 nm) despite appearing as white on the surface [25].

Animal experiments have indicated that a high-fat diet, combined with ten weeks of blue light exposure, could result in greater body weight, hyperlipidemia, and reduced insulin sensitivity compared to the control group, leading to metabolic abnormalities [10]. In addition, a high-fat diet combined with long-term exposure to blue light (24 h, 12 weeks, 150 lux) can decrease the total antioxidant capacity in mice and may exacerbate renal tissue damage by affecting the Nrf2/HO-1 signaling pathway and activating pro-inflammatory cytokines [9]. In our study, although blue light exposure did not significantly affect body weight or the lipid metabolic abnormalities induced by the HFHF diet during the experiment, we observed a higher NAS score and more pronounced Nrf-2 and NF-κB expression in the pathological sections. Furthermore, the analysis also revealed that the HB group indeed had higher hepatic MDA and inflammatory cytokine concentrations. When dietary factors (such as excessive fat) and environmental factors lead to an imbalance between ROS and the antioxidant system in vivo, the body needs to rely on its own antioxidant system to clear excessive free radicals to maintain physiological stability [27]. Nrf2 is an important upstream transcription factor that regulates redox reactions, and it is typically stable in the cytoplasm and bound to the Kelch-like ECH-associated protein 1 (Keap1). When the internal environment changes and ROS increases, Nrf2 dissociates from Keap1, resulting in the activation and translocation of Nrf2 into the nucleus, where it interacts with the antioxidant response element, and this subsequently activates downstream antioxidant enzymes to reduce ROS generation in the body, regulating oxidative stress and reducing lipid peroxidation [4]. These results suggest that the combination of HFHF and blue exposure may indeed cause an increase in hepatic oxidative stress and inflammation, thereby upregulating the expression of Nrf2 in response to tissue damage.

On the other hand, some studies have also found that blue light exposure may cause gut microbiota dysbiosis and potentially lead to neuroinflammation by affecting the MyD88/NFκB pathways [8]. Gut dysbiosis has a significant impact on the progression of diet-induced fatty liver disease. The composition of the human gut microbiota is diverse, and numerous recent studies have suggested that the gut microbiota may influence physiological functions and even be associated with various clinical symptoms [28]. For example, an imbalance between *Firmicutes* and *Bacteroidetes* is considered to be associated with various metabolic diseases [29]. When excessive endotoxin in the intestinal lumen passes through the intestinal mucosa and enters the bloodstream, it activates the TLR4 signaling pathway in the liver. This process proceeds through the downstream MyD88 pathway, resulting in the activation of NF-κB and the increased secretion of pro-inflammatory cytokines, such as TNF-α and IL-1β, which results in liver inflammation. Furthermore, TLR4 can also act through another downstream pathway, TRIF, which in addition to potentially activating the expression of interferon (IFN)-related factors via interferon regulatory factor 3 (IRF3), also causes the activation of the common downstream NF-κB signal transduction, further promoting tissue inflammation and damage [29]. Although some animal studies have suggested that a long-term high-fat or high-fructose diet may result in gut microbiota imbalances, thereby affecting normal physiological functions [4,5], we did not observe a difference in the TLR4 signaling pathway among groups. These results suggest that the effect of this study may not be through this pathway, or the experimental conditions are still insufficient for influencing this pathway.

In the gut microbiota analysis, we found that both analysis methods showed significantly changed genera, including Bacteroides, Corynebacterium, and Muribaculum, reflecting the impact of a high-fat diet and blue light exposure on the gut microbial composition. The genus Bacteroides belongs to the phylum Bacteroidetes, order Bacteroidales, and family Bacteroidaceae, and it is one of the main dominant bacterial groups in the mammalian gut. This genus plays a crucial role in energy metabolism, the breakdown of complex carbohydrates, and host immune regulation, and it is considered a core bacterium for maintaining intestinal stability [30]; however, the effects of different species may vary, and some strains may also be pathogenic [31]. In this study, we found that the relative abundance of Bacteroides was increased in both the H and the HB groups, suggesting that Bacteroides may exhibit a compensatory growth advantage under an HFHF diet; however, the functional differences at the species level still require further clarification. Most Corynebacteria are commonly found on the skin, but they have also been detected in gut samples recently, and some strains are believed to be involved in lipid metabolism and cholesterol conversion [32]. The present study showed that the presence of this genus was significantly higher in the HB group, suggesting that blue light exposure might affect this bacterium under the HFHF background; however, further experiments are needed to verify its resulting physiological function. The genus Muribaculum has been reported to be able to decompose dietary fiber and polysaccharides, producing short-chain fatty acids (SCFAs), which suggests that it may play a crucial role in maintaining the intestinal barrier, immune stability, and energy balance [33]. Muribaculum was found to be lower in both the H and HB groups in our study. These results suggest that both a long-term HFHF diet and changes in the blue light environment may weaken the dominant gut bacteria that metabolize fiber, thereby affecting overall microecological stability and energy metabolism.

In this study, we obtained interesting results that differ from previous research on the potential eye damage caused by blue light. In addition to consuming an unhealthy diet that is high in fat and sugar, daily exposure to a certain amount of blue light may have the potential to increase oxidative and inflammatory responses in the body, which may accelerate the progression of liver disease. However, the present study still has some limitations. For example, considering the acceptance of blue light stimulation, we prioritized selecting an albino animal strain, but this strain may not be the most sensitive choice for liver or metabolic disorders. Although the daily blue light exposure time in this experiment was not as long as in other experiments, the possible impact on the circadian rhythm still cannot be completely ruled out. Additionally, since neither the diet nor the blue light exposure dose in this experiment is considered to cause acute damage, the study duration may not be long enough, and the sample size for gut microbiota analysis may have affected statistical power. A longer experimental period might be needed to observe more pronounced results. Due to changes in modern living environments and dietary patterns, many metabolic abnormalities have arisen. Besides diets rich in high-fat and high-sugar foods, the effects of extensive blue light exposure in modern life cannot be ignored. Therefore, understanding the potential combined effects and mechanisms under conditions that involve both dietary and environmental factors is crucial for developing future prevention and improvement strategies, as well as for furthering clinical research. Our present study has established preliminary data, and future studies may consider more related issues to further clarify the underlying mechanisms.

## 5. Conclusions

In conclusion, although 16-week exposure to blue light alone did not show a significant impact on liver injury, concurrent exposure to blue light and an unhealthy dietary pattern may increase hepatic oxidative stress and inflammatory responses, alter the gut microbiota composition, and potentially accelerate the progression of fatty liver disease.

Blue light exposure is a health factor that is difficult to avoid in modern life. Although the current understanding of its influence on the progression of liver disease is still limited, blue light exposure remains a highly concerning issue that is worth noting and warrants further investigation. This study could serve as a reference for further advanced and clinical research studies.

## Figures and Tables

**Table 1:** Liquid diet composition (g/L).

g/L	C Diet	HFHF Diet
casein	82	82
L-cystine	1.6	1.6
corn oil	75	155.5
dextrin maltose	247	-
fructose	-	66
AIN-93M Vitamin	10	10
AIN-93M Mineral	30	30
cholesterol	-	1.15
cholic acid	-	0.75
choline bitartrate	1.1	0.75
cellulose	20	20
xanthan gum	6	6

Casein (high nitrogen), fructose, mineral mixture (AIN-93M mineral mixture), cellulose (non-nutritive bulk), and vitamin mixture (AIN-93M vitamin mixture) were purchased from MP Biomedicals (Santa Ana, CA, USA). Dextrin maltose was from Ingredion Incorporated (Westchester, IL, USA). Cholesterol, choline bitartrate, cholic acid and xanthan gum were from Sigma.

**Table 2:** Body weight, food intake and liver weight of mice at the end of the study.

	Male		Female		OVX		Interaction *p*-Value
	C	BL	C	BL	C	BL	
BW (g)	39.7 ± 1.8	39.9 ± 1.5	30.4 ± 1.7 ^#^	31.0 ± 2.4 ^†^	33.7 ± 1.9 ^#^	32.2 ± 1.6 ^†^	0.3323
Food intake (g/d)	6.0 ± 0.1	6.1 ± 0.1	5.5 ± 0.3	5.2 ± 0.1 ^†^	4.7 ± 0.3 ^#^	4.5 ± 0.4 ^†^	0.1244
Calorie intake (kcal/d)	25.2 ± 0.4	25.6 ± 0.3	23.1 ± 1.2	21.8 ± 0.6 ^†^	19.8 ± 1.2 ^#^	18.8 ± 1.6 ^†^	0.2233
Liver							
Weight (g)	1.9 ± 0.2	1.8 ± 0.3	1.5 ± 0.2 ^#^	1.4 ± 0.3 ^†^	1.3 ± 0.0 ^#^	1.3 ± 0.2 ^†^	0.9662
LW/BW (%)	4.5 ± 0.5	4.2 ± 0.8	4.6 ± 0.6	4.4 ± 0.4	3.9 ± 0.2	4.0 ± 0.5	0.6864

Values represent means ± SD; *n* = 6 per group. Data were analyzed using two-way ANOVA with Tukey’s post hoc test. ^#^ Significantly different from the C group of females or OVX compared with the male control group (C (male) vs. C (female), C (male) vs. C (OVX), *p* < 0.05). ^†^ Significantly different from the BL group of females or OVX compared with the male BL group (BL (male) vs. BL (female), BL (male) vs. BL (OVX), *p* < 0.05). C, control group; BL, blue light exposure group; BW, body weight; LW, liver weight.

**Table 3:** Weight, serum biochemical, and hepatic lipid parameters at the end of the study.

	C	H	HB	*p*-Value
BW (g)	44.8 ± 4.0	48.2 ± 7.8	44.8 ± 6.6	0.5717
Food intake (g/d)	9.7 ± 0.8	10.2 ± 1.3	10.6 ± 0.8	0.5640
Calorie intake (Kcal/d)	19.3 ± 1.7	21.5 ± 1.0	21.8 ± 1.0	0.5480
Blood				
glucose (mg/dL)	131.7 ± 25.6	129.5 ± 35.6	157.7 ± 27.5	0.2687
TC (mg/dL)	225.8 ± 30.2	181.3 ± 52.3	166.7 ± 33.5	0.0773
TG (mg/dL)	127.9 ± 24.3	47.5 ± 161 *	39.2 ± 8.4 *	<0.0001
HDL-C (mg/dL)	213.3 ± 28.5	167.1 ± 41.5	153.3 ± 28.9 *	0.0285
LDL-C (mg/dL)	41.7 ± 10.6	59.2 ± 15.1	59.2 ± 17.6	0.1222
TC/HDL-C	1.06 ± 0.04	1.07 ± 0.05	1.08 ± 0.02	0.5833
AST (U/L)	84.0 ± 8.4	133.8 ± 45.5	160.4 ± 59.6 *	0.0420
ALT (U/L)	53.8 ± 6.2	176.7 ± 84 *	165.0 ± 73.4 *	0.0170
Cre (mg/dL)	0.37 ± 0.04	0.35 ± 0.05	0.36 ± 0.05	0.8209
Liver				
LW (g)	1.9 ± 0.2	2.6 ± 0.5 *	2.1 ± 0.2	0.0157
LW/BW (%)	4.3 ± 0.3	5.4 ± 0.8 *	4.7 ± 0.4	0.0087
TC (µmol/g Liver)	0.8 ± 0.3	1.1 ± 0.4	0.6 ± 0.2	0.0733
TG (µmol/g Liver)	3.8 ± 1.9	4.8 ± 3.4	4.3 ± 0.7	0.7589
White adipose tissue				
mWAT (g)	0.9 ± 0.2	0.8 ± 0.5	0.6 ± 0.4	0.5639
prWAT (g)	1.1 ± 0.4	2.1 ± 0.9	1.9 ± 1.2	0.1753
eWAT (g)	2.0 ± 0.9	0.9 ± 0.5 *	0.8 ± 0.6 *	0.0102
AF (g)	4.0 ± 1.3	3.8 ± 1.8	3.4 ± 2.2	0.8657
AF/BW (%)	8.8 ± 2.3	7.6 ± 2.5	7.2 ± 3.6	0.6210

Values represent means ± SD; *n* = 6 per group. Data were analyzed using one-way ANOVA with Tukey’s post hoc test. * Significantly different from the control group (C vs. H or HB, *p* < 0.05). C, control diet; H (HFHF), high-fat-high-fructose diet; HB, HFHF diet + blue light exposure group. BW, body weight; LW, liver weight; TC, total cholesterol; TG, triglyceride; HDL-C, high-density lipoprotein cholesterol; LDL-C, low-density lipoprotein cholesterol; AST, aspartate transaminase; ALT, alanine transaminase; Cre, creatinine; mWAT, mesenteric white adipose tissue; prWAT, perirenal WAT; eWAT, epididymal WAT; AF, abdominal fat was calculated as mWAT + prWAT + eWAT.

## Data Availability

Data are contained within the article.
